# *In vitro* Evaluation of the Anti-hypercholesterolemic Effect of *Lactobacillus* Isolates From Various Sources

**DOI:** 10.3389/fmicb.2022.825251

**Published:** 2022-02-28

**Authors:** Raymond Rubianto Tjandrawinata, Medicia Kartawijaya, Apriliana Wahyu Hartanti

**Affiliations:** ^1^Dexa Laboratories of Biomolecular Sciences, PT Dexa Medica, Cikarang, Indonesia; ^2^Faculty of Biotechnology, Atma Jaya Catholic University of Indonesia, Jakarta, Indonesia

**Keywords:** *Lactobacillus*, probiotic, cholesterol removal, *in vitro*, anti-cholesterol

## Abstract

The anti-hypercholesterolemic effect of 11 *Lactobacillus* isolates was investigated *in vitro* by measuring remaining cholesterol in growth media, growth ability in media supplemented with cholesterol, and BSH activity. Among the selected isolates, DLBSH104, DLBSH122, and DLBSK207 have demonstrated outstanding potential as cholesterol-lowering cultures. The three isolates showed high cholesterol removal by growing cells, whereas resting and dead cells showed less cholesterol removal. Furthermore, visualization of those isolates in growing and non-growing states by SEM showed the ability of DLBSH104 to attach cholesterol to their cell surface. In contrast, alteration of DLBSH122 and DLBSK207 cells did not involve surface attachment of cholesterol. Thus, the isolates’ ability to remove cholesterol is mainly attributed to the cells’ metabolically active state that assimilates and incorporates cholesterol into the cell membrane as reflected by a significantly higher cholesterol removal in a growing state than a non-growing state. Only in DLBSH104 did cholesterol removal also involve attachment on the cell surface. Moreover, DLBSH104 has beneficially affected the host cell by a significant reduction of NPC1L1 mRNA levels that are responsible for intestinal cholesterol absorption. In hepatic cells, cell-free supernatant (CFS) from DLBSH104 and DLBSK207 were able to reduce LDLR and HMGCR mRNA at the transcription level. To sum up, *L. helveticus* DLBSH104 and *L. plantarum* DLBSK207 are confirmed as isolates with an anti-hypercholesterolemic effect.

## Introduction

Dyslipidemia is one of the most significant CVD risk factors (Kopin and Lowenstein, 2017), and uncontrolled hyperlipidemia could lead to atherosclerosis and, eventually, cardiac arrest (Artham et al., 2008). Therefore, intensive efforts have been made to develop lipid-lowering drugs such as statin (Vaughan et al., 1996). To date, seven HMG-CoA reductase inhibitors (statins), namely, lovastatin, simvastatin, pravastatin, atorvastatin, rosuvastatin, fluvastatin, and pitavastatin, are approved and used as the first-line drugs for hypercholesterolemia (Vaughan et al., 1996; Khvorova, 2017). However, side effects of statins consumption such as dysregulation of glucose metabolism (Ferri and Corsini, 2020), myopathy (the symptom of which are muscle weakness and muscular pain), and rhabdomyolysis are reported (Bellosta et al., 2004). Therefore, the search for natural and safe therapeutic agents from plants (Kartawijaya et al., 2016; Najib et al., 2018), animals (Sinaga et al., 2020; Pinzon et al., 2021), and probiotics (Bhat and Bajaj, 2019; Khare and Gaur, 2020) has been conducted to prevent and lower the burden of dyslipidemia.

Supplementation of probiotics with cholesterol-lowering ability has been proposed as a useful strategy to reduce serum total cholesterol (Wang et al., 2018). Several studies have reported the evaluation of potential probiotics with cholesterol-lowering ability that showed positive effects in *in vitro* and *in vivo* studies and human volunteers (Sivamaruthi et al., 2019). It was reported for the first time that the consumption of *L. acidophilus* fermented milk reduced serum cholesterol in hyperlipidemia in human subjects in 1974 (Mann, 1974). Since then, many probiotic strains have developed as a biotherapeutics substance for dyslipidemia (Fuentes et al., 2016; Wang et al., 2018; Sivamaruthi et al., 2019). Various mechanisms of probiotics-mediated cholesterol reduction have been proposed. Among them is bile salt hydrolase (BSH) activity. Deconjugation of bile acids by BSH activity of certain microbes produces free amino acids and secondary bile acids that are less soluble and will be excreted in feces (Choi and Chang, 2015; Bhat and Bajaj, 2019). Another well-known mechanism is the attachment on the cell surface and the incorporation of cholesterol into the bacterial membrane-phospholipid bilayer. Attachment of cholesterol on the bacteria cell surface results in a smaller number of readily absorbed cholesterol in the intestine (Lye et al., 2010; Choi and Chang, 2015). In addition, cholesterol assimilation during bacterial growth, cholesterol precipitation with a deconjugated bile salt, and conversion of cholesterol to 5β-coprostanol also contribute to the cholesterol removal in the intestine (Bhat and Bajaj, 2019; Khare and Gaur, 2020). In recent years, probiotics were also reported to interact with host cells in managing hypercholesterolemia through molecular mechanisms such as downregulation of NPC1L1, a gene responsible for cholesterol uptake in the intestine (Huang and Zheng, 2010; Le and Yang, 2019).

In the current study, we evaluated the capability of 11 *Lactobacillus* isolates as a cholesterol-lowering agent through several *in vitro* assays. We aimed to obtain specific *Lactobacillus* isolates that are potentially lowering blood serum cholesterol since solid evidence was found that probiotic efficacy is strain-specific (Campana et al., 2017; de Melo Pereira et al., 2018; McFarland et al., 2018). The most beneficial isolates will be investigated further to develop a hypercholesterolemic probiotic product.

## Materials and Methods

### Bacterial Isolates and Growth Condition

Eleven *Lactobacillus* isolates used in this study were obtained from the Metabolic Engineering Section of Dexa Laboratories of Biomolecular Science. All cultures have been isolated from various natural sources, characterized for their probiotic’s potential and safety (*in vitro*, data not shown), and identified by 16S rRNA gene sequencing. The isolation source and the 16S rRNA identification of each isolate can be seen in Table 1. All isolates were maintained in 20% glycerol stock at −80°C and sub-cultured twice prior to the experiments. In addition, the organisms were grown in *Lactobacillus* MRS HiVeg Broth (HiMedia; MV369) at 37°C for 18–22 h before experiments. Three independent experiments were performed in duplicate for each assay throughout the study.

**TABLE 1:** List of *Lactobacillus* isolates used in this study.

Isolation source	Codes	16s rRNA identification	Accession number
Human breast milk	DLBSA201	*Lactobacillus helveticus*	OL989228
	DLBSA202	*Lactobacillus helveticus*	OM004046
	DLBSL101	*Lactobacillus helveticus*	OM004034
	DLBSL102	*Lactobacillus helveticus*	OM021854
	DLBSL103	*Lactobacillus helveticus*	OM004045
Mango (*Mangifera indica*) fruit	DLBSH113	*Lactiplantibacillus plantarum*	OM004007
	DLBSH104	*Lactobacillus helveticus*	OM004009
	DLBSH122	*Lactiplantibacillus plantarum*	OM004015
	DLBSH131	*Lactiplantibacillus plantarum*	OM004018
	DLBSH235	*Lactobacillus helveticus*	OM004019
Goat colostrum	DLBSK207	*Lactiplantibacillus plantarum*	OM004020

### Cell Culture

Human colorectal adenocarcinoma (Caco-2) and human hepatocarcinoma (HepG2) cell lines were purchased from ATCC. Caco-2 was grown in MEM medium (Gibco; 61100-061) containing 10% (v/v) FBS (Gibco; 26140087) and 1% (v/v) Penicillin–Streptomycin (Gibco; 15140-122) while HepG2 was grown in MEM-alpha (Gibco; 12000-022) containing 10% (v/v) FBS and 1% (v/v) Penicillin–Streptomycin. All cells were maintained in 5% CO_2_ at 37°C. Caco-2 and HepG2 cells were plated at a 6-well plate with a density of 3 × 10^6^ cells/well for assays. The Caco-2 cells were incubated for 18–21 days after confluence to produce the monolayer of Caco-2 cells and the culture media was changed every 2 days. Two hours before experiments, the media was changed to a serum-starvation media containing 0.5% (v/v) FBS without antibiotics.

### Cholesterol Removal by Growing, Resting, and Heat-Killed Cell

One percent (1%) of sub-cultured *Lactobacillus* isolates were inoculated into MRS broth (10 ml) containing 0.3% (b/v) ox gall (Oxoid; LP0055) and 100 mg/L water-soluble cholesterol (Sigma Aldrich; C4951) to prepare cholesterol removal assay by growing cells. For resting cell preparation, 1% of sub-cultured *Lactobacillus* isolates that were previously washed 3 × with PBS were suspended in 0.05 M phosphate buffer (pH 6.8) supplemented with 0.3% (w/v) ox gall and 100 mg/L cholesterol. Heat-killed cells were prepared from saline-suspended cell pellets of sub-cultured *Lactobacillus* isolates that were heated at 121°C for 15 min before being added into MRS broth containing 0.3% ox gall and 100 mg/L cholesterol. Incubation was done at 37°C for 20 h in all assays, after which the mixture was centrifuged at 13,000 rpm for 5 min at 4°C. MRS broth with and without the addition of 0.3% ox gall (b/v) and 100 mg/L water-soluble cholesterol were used as a negative and positive control, respectively. The remaining cholesterol in the supernatant was measured using Cholesterol Quantitation Kit (Sigma Aldrich; MAK048).

### Bile Salt Hydrolase Activity

BSH activity assay was conducted as previously described by Choi and Chang (2015) with modification. Briefly, 10 μl of overnight grown *Lactobacillus* isolates were spotted onto MRS agar containing 0.5% (w/v) ox gall and 0.37 g/L of CaCl_2_ and then were incubated anaerobically at 37°C for 72 h. Plates were observed at the end of incubation time and were categorized as an isolate with no precipitation ability (–), low precipitation ability (+), medium precipitation ability (+ +), and strong precipitation ability (+ + +).

### Growth in Bile Acids and Cholesterol

The tolerance ability of the selected *Lactobacillus* isolates to survive in the presence of bile salt and cholesterol was studied by inoculating about 1 × 10^9^ CFU of overnight *Lactobacillus* into MRS broth supplemented with 0.3% ox gall and 100 mg/L water-soluble cholesterol for 24 h at 37°C. *Lactobacillus* growth in MRS broth without ox gall and cholesterol was used as control. After incubation, all isolates were plated in MRS agar to calculate the number of bacterial growths. The ability of *Lactobacillus* isolates to grow under bile acids and cholesterol was described as log reduction between *Lactobacillus* growth in control and ox gall/cholesterol-supplemented media.

### Scanning Electron Microscopy

For this experiment, growing, resting, and heat-killed cells were prepared as described in section “Cholesterol Removal by Growing, Resting, and Heat-Killed Cell,” and *Lactobacillus* isolates that grew in MRS broth without any supplementation were used as a control. After incubation at 37°C for 24 h, cells were pelleted through centrifugation at 13,000 rpm for 5 min at 4°C. Before being fixed in 10% formaldehyde overnight at 4°C, cell pellets were washed twice with PBS (pH 6.8). Serial dehydration was done by ethanol 30, 40, 50, 60, 70, 80, and 96% for 2–3 min each. Specimens were coated with gold and then were visualized by using Scanning Electron Microscopy (SEM) microscope JEOL model JSM 6510 at a magnification of × 10.000.

### Cytotoxicity of *Lactobacillu*s Candidates on Caco-2 Cell

Potential *Lactobacillus* isolates with a concentration between 1 × 10^6^ and 1 × 10^9^ CFU/ml were added into 1 × 10^4^ cells/well of Caco-2 in a 96-well plate. Before the addition, overnight *Lactobacillus* isolates were washed twice with PBS (pH 6.9) at 13,000 rpm, 4°C for 5 min, and then were diluted in MEM medium. Cell cytotoxicity assay was performed after incubation for 24 h at 37°C using Vybrant MTT Proliferation Assay Kit (Thermo Fischer Scientific; V13154). Briefly, 10 μl of the 12 mM MTT stock solution was added to each well and the mixture was incubated at 37°C for 4 h. Fifty microliters of DMSO was added to dissolve the formazan crystal formed at the end of the reaction before the absorbance was read at 540 nm.

### Effects of *Lactobacillus* on Cholesterol Metabolism-Related Genes in Caco-2 Cells

Overnight potential *Lactobacillus* isolates were centrifuged at 13,000 rpm for 5 min at 4°C to obtain a cell pellet. The cell pellets were washed twice in sterile PBS (pH 6.8) before re-suspending in antibiotic-free MEM with 0.5% FBS. The cell number was adjusted to 1 × 10^7^ CFU/ml before addition to monolayer Caco-2 cells. Prior to co-incubation experiments, Caco-2 cells were washed three times with warm sterile PBS to remove antibiotics. The number of bacteria added for co-incubation experiments with Caco-2 cells was adjusted to 1 × 10^6^–1 × 10^9^ CFU/ml after being washed three times with phosphate buffer saline (PBS). Water-soluble cholesterol was added into all wells, including control, with a 70 μg/ml concentration. RNA extraction was conducted after co-incubating Caco-2 with LAB strains for 24 h at 37°C with 5% CO_2_. The mRNA expression levels from this experiment were measured by qRT-PCR.

### Cholesterol Regulation in HepG2 Cells

HepG2 cells were seeded 24 h before treatment in a 6-well plate. Two hours prior to the addition of CFS with concentrations of 10–30% and 70 μg/ml water-soluble cholesterol, the media was changed to serum starvation media containing 0.5% FBS without antibiotics. Incubation was carried out at 37°C with 5% CO_2_ for another 24 h before RNA extraction and quantification by qRT-PCR. CFS for this experiment was obtained by centrifugating the overnight *Lactobacillus* isolates at 13,000 rpm, 4°C for 5 min. CFS was then stored at −80°C after sterilizing by using a 0.45-μm membrane filter until used.

### Detection of Cholesterol Metabolism-Related Genes Expression by Real-Time Quantitative PCR

At the end of experiments, Caco-2 and HepG2 cells were washed three times with PBS, and the total RNA was isolated using Trizol reagent. According to the manufacturer’s protocol, 1 μg of total RNA was synthesized into cDNA by ReverTra Ace^®^ qPCR RT Master Mix. RT-qPCR was prepared using KAPA SYBR^®^ FAST qPCR on a CFX96 sequence detection system (Biorad, Hercules, CA, United States). Primers for housekeeping and target genes were shown in Table 2, and all primer sets were confirmed by agarose gel electrophoresis before being used. PCR was performed in the following condition: denaturation at 95°C for 3 min, annealing at 58°C for 30 s, extension at 72°C for 1 min, and final extension at 72°C for 5 min. β-actin was used to normalize the gene expression values and the relative mRNA levels were calculated by 2^–ΔΔ^ ^Ct^.

**TABLE 2:** List of primer used for RT-qPCR.

Target genes	Sequences (5′–3′)	Tm (°C)	References
β-actin	F: ACTCTTCCAGCCTTCCTTCC R: CGTACAGGTCTTTGCGGATG	58	Michael et al., 2016
NPC1L1	F: TCTTCCCCTTCCTTGCCATT R: CGGCAGGGTAATTGTTGAGG	58	Michael et al., 2016
ABCG5	F: CTCTTGTGCTACTTGGTATCGTC R: CTGCCACAAGTGAAATTCAGTCC	58	Yang et al., 2018
ABCG8	F: ATCGGCTACCCCTGTCCTC R: GTCCTCGTCAAGATCCTTCGT	58	Yang et al., 2018
HMGCR	F: AGTTTGAAGAGGATGTTTTG R: TCCCTTACTTCATCCTGTGA	58	Tandrasasmita et al., 2021
LDLR	F: CTGTCGTGTGTGTTGGGAT R: CGACAAGATTGGGGAAGTG	58	Berlian et al., 2018

*Note: NPC1L1, Niemann-pick C1-like 1; ABCG5, ATP Binding Cassette Subfamily G Member 5; ABCG8, ATP Binding Cassette Subfamily G Member 8; HMGCR, 3-Hydroxy-3-Methylglutaryl-CoA Reductase; LDLR, low-density lipoprotein receptor.*

### Statistical Analysis

The data were analyzed using SPSS version 25 (Statistical Package for the Social Sciences; SPSS Inc., Chicago, IL, United States). One-way analysis of variance (ANOVA) followed by Duncan’s multiple comparisons test with a significance level at α = 0.05 was used to see the statistical difference between groups. Significance between the two groups was determined by Student’s *t*-test with *p*-value < 0.05 considered statistically significant.

## Results

### Cholesterol Removal by *Lactobacillus* Isolates *in vitro*

**FIGURE 1:**
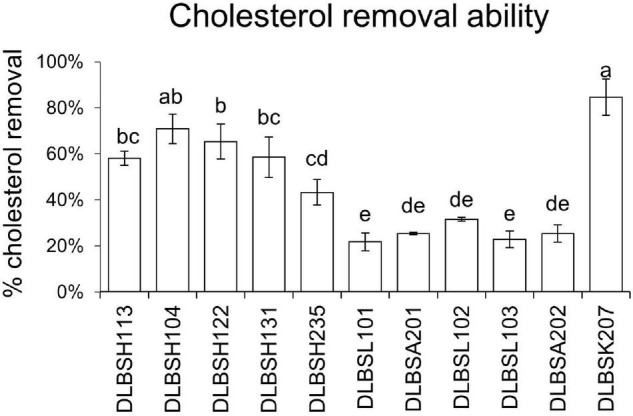
Cholesterol removal ability of DLBS’ probiotic isolates collection. Cholesterol removal ability from 11 different isolates was measured after incubation at 37°C for 24 h in MRS broth media + 0.3% ox gall and cholesterol (100 mg/L). The result is expressed as mean ± SD of at least three independent experiments. Different letters (a–e) represent significant differences (*p* < 0.05) for the comparison of all groups by one-way ANOVA.

The removal of cholesterol by 11 *Lactobacillus* isolates grown in MRS broth supplemented with 0.3% ox gall is shown in Figure 1. The ability of *Lactobacillus* isolates to remove cholesterol from media ranged from 21.72 to 84.67% after 20-h incubation. Specifically, isolate DLBSK207 has the highest ability to remove cholesterol (84.67%) followed by isolate DLBSH104 (70.88%), DLBSH122 (65.34%), DLBSH131 (58.55%), and DLBSH113 (58.10%).

### Bile Salt Hydrolase Activity

The ability of *Lactobacillus* isolates to hydrolyze BSH was tested using MRS agar with 0.5% ox gall. The various abilities of *Lactobacillus* isolates to precipitate ox gall in media were found after 3 days of incubation (Table 3). In particular, DLBSH122 and DLBSH131 showed a solid ability to precipitate bile acid, while DLBSH113, DLBSH235, and DLBSK207 showed moderate BSH activity. Furthermore, DLBSH104 showed low BSH activity, and DLBSL101, DLBSL102, DLBSL103, DLBSA201, and DLBSA202 showed no BSH activity.

**TABLE 3:** BSH Activity and Growth of *Lactobacillus* in the presence of 0.3% oxgall and cholesterol (100 mg/L).

Strain	BSH activity*	Log reduction of *Lactobacillus* growth in MRS Broth + 0.3% oxgall and cholesterol (100 mg/L) **
DLBSH113	++	3.3 ± 0.42^a^
DLBSH104	+	1.48 ± 0.18^c^
DLBSH122	+++	1.1 ± 0.17^d^
DLBSH131	+++	3.14 ± 0.32^a^
DLBSH235	++	3.09 ± 0.37^a^
DLBSL101	–	2.77 ± 0.19^b^
DLBSA201	–	3.22 ± 0.38^a^
DLBSL102	–	2.58 ± 0.08^b^
DLBSL103	–	2.67 ± 0.09^b^
DLBSA202	–	2.63 ± 0.13^b^
DLBSK207	++	1.24 ± 0.16^cd^

* BSH activity was expressed based on the ability to precipitate bile salt on BSH screening medium: (–) no precipitation; (+) low BSH activity (+ +) medium BSH activity; (+ + +) strong BSH activity.

** All values are means ± standard deviation from 3 independent experiments. Different letters (a–d) represent significant differences (p < 0.05) for the comparison of all groups by one-way ANOVA.

### Bile Acid and Cholesterol Effects on *Lactobacillus* Growth

MRS broth supplemented with bile acid (0.3% ox gall) and cholesterol (100 mg/L) was used in this assay. The growth of each isolate in media without bile acid and cholesterol was used as a control. Generally, all isolates’ growth was inhibited in the presence of ox gall and cholesterol (Table 3). The highest log reduction was found in isolate DLBSH113 (3.3 ± 0.42), DLBSH131 (3.14 ± 0.32), and DLBSH235 (3.09 ± 0.37), while isolate DLBSH122 (1.1 ± 0.17), DLBSK207 (1.24 ± 0.16), and DLBSH104 (1.48 ± 0.18) grew well despite the addition of bile acid and cholesterol. In addition, the correlation between the ability of *Lactobacillus* isolates to grow in cholesterol and ox gall supplemented media with BSH was not observed. DLBSH131 and DLBSH235, which showed solid and moderate BSH activity, demonstrated a high log reduction in cholesterol/ox gall supplemented MRS broth (3.14 ± 0.42 and 3.09 ± 0.37, respectively). In contrast, DLBSH104 exhibited low log reduction (1.48 ± 0.18), although it has moderate BSH activity.

### Cholesterol Removal by Growing and Non-growing Cells of *Lactobacillus* Isolates

**FIGURE 2:**
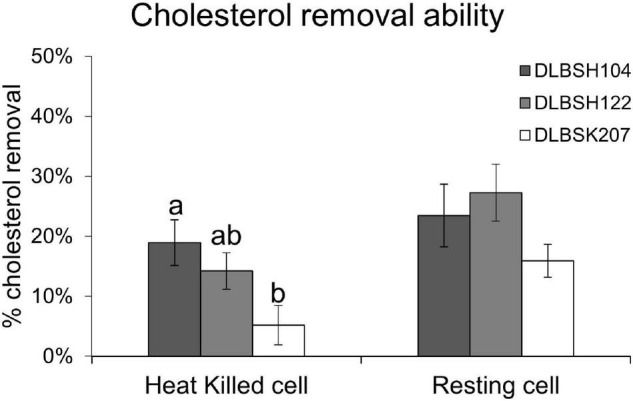
Cholesterol removal ability of potential *Lactobacillus* strains in growing and non-growing state. Cholesterol removal ability of growing, resting, and heat-killed cells of 3 potential *Lactobacillus* strains was measured after incubation at 37°C for 24 h in media + 0.3% ox gall and cholesterol (100 mg/L). The result is expressed as mean ± SD of three independent experiments. Different letters a,b represent significant differences (*p* < 0.05) for the comparison of all groups by one-way ANOVA.

Three *Lactobacillus* isolates, *L. helveticus* DLBSH104, *L. plantarum* DLBSH122, and *L. plantarum* DLBSK207, were selected for further experiments. Previous studies (Lye et al., 2010; Miremadi et al., 2014) confirmed that resting and dead cells of probiotics still have the ability to remove cholesterol by adhering to the cholesterol on their cell surface. This study examined the ability of resting and heat-killed cells of our three potential isolates to remove cholesterol. Cholesterol removal by resting and heat-killed cells of *Lactobacillus* ranged from 15.92 to 27.24% and from 5.17 to 18.93%, respectively. Thus, both resting and heat-killed cells of those isolates could remove cholesterol from the media. However, their removal ability in the non-growing state was significantly lower compared to their growing state (Figure 2). In addition, the cholesterol removal ability of DLBSH122 and DLBSK207 in the resting state was significantly higher than its heat-killed state, although this difference was not observed in DLBSH104 (Figure 2).

### Scanning Electron Microscope Observation

**FIGURE 3:**
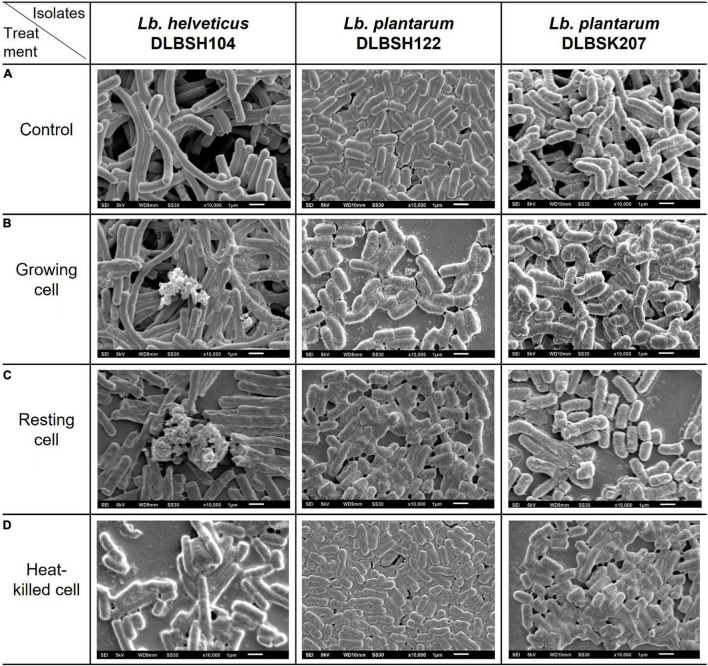
Scanning electron micrograph of potential probiotics in media containing no cholesterol **(A)** and media containing cholesterol **(B–D)** after incubated at 37°C for 20 h.

In our present study, the cell surface of DLBSH104 has the capability to attach cholesterol, although only a tiny amount of cholesterol has adhered in heat-killed cells (Figure 3). In contrast, a much smaller amount of cholesterol was found on the cell surface of DLBSH122 and DLBSK207, but there was an alteration in their cell morphology shape as indicated by a bigger and thicker cell size compared to control (Figure 3). Furthermore, shorter cell length was observed in DLBSK207 cells when incubated in a media containing cholesterol. In addition, morphological changes of DLBSH122 and DLBSK207 were mainly detected in growing and resting state, but in heat-killed cells, the shape was relatively similar to control. These data suggest that cholesterol may be assimilated and incorporated into DLBSH122 and DLBSK207 cell membrane during the metabolic process.

### Effects of *Lactobacillus* Isolates Toward Cholesterol Metabolism-Related Genes in Human Intestinal Epithelial Cells (Caco-2)

**FIGURE 4:**
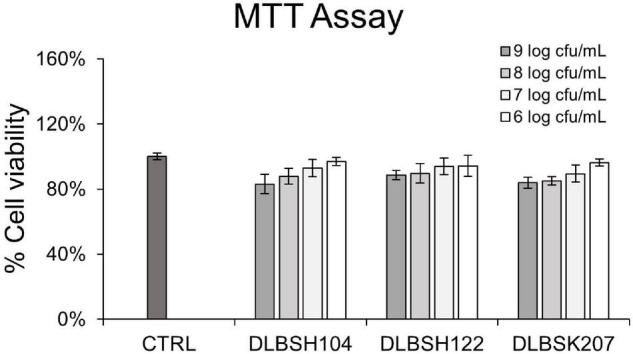
Cell viability of Caco-2 treated with potential *Lactobacillus* strains for 20 h. Caco-2 was treated with 3 potential LAB strains with several concentrations (1 × 10^6^–1 × 10^9^ CFU/ml). Then, the cell viability of Caco-2 after treatment was assessed by MTT assay. The result is expressed as mean ± SD of at least three independent experiments. Statistical analysis was determined by using one-way ANOVA.

MTT assay was performed to evaluate the cytotoxicity effect of DLBSH104, DLBSH122, and DLBSK207 in Caco-2 cells. The addition of 6–9 log CFU/ml of *Lactobacillus* isolates did not significantly affect Caco-2 cells viability (Figure 4). Less than 10 and 20% of lower Caco-2 cell viability were observed when 6–7 log CFU/ml or 8–9 log CFU/ml probiotics were added, respectively (Figure 4).

**FIGURE 5:**
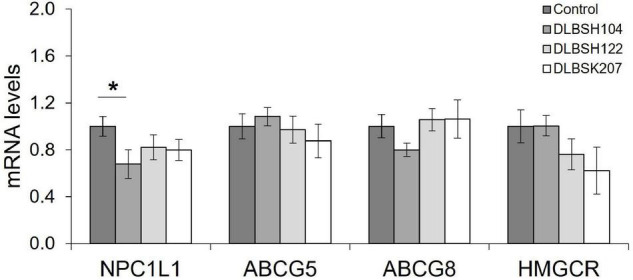
Transcript levels of NPC1L1, ABCG5, ABCG8, and HMGCR of Caco-2 treated with cholesterol (70 μg/L) and potential *Lactobacillus* strains (10^7^ CFU/ml). mRNA levels of several cholesterol homeostasis genes were measured after cholesterol and LAB treatment at 37°C for 20 h. The result is expressed as mean ± SD of at least three independent experiments. Student’s *t*-test was used and * represents a significant difference with a *p*-value < 0.05 compared to the control.

Next, we investigated the cholesterol-lowering activities of our isolates through mRNA expression of cholesterol homeostasis-related genes in the intestine (NPC1L1, ABCG5, ABCG8, and HMGCR). All isolates could reduce the expression levels of NPC1L1, although only DLBSH104 significantly reduced the expression levels of NPC1L1 (*p* = 0.03) while DLBSK207 (*p* = 0.06) and DLBSH122 (*p* = 0.11) reduced NPC1L1 expression mildly (Figure 5). In contrast, there were no significant changes in the expression of ABCG5, ABCG8, and HMGCR genes in Caco-2 cells. However, there was a reduction trend of the HMGCR gene expression after Caco-2 cells were treated with DLBSH122 (*p* = 0.12) and DLBSK207 (*p* = 0.08).

### Regulation of *Lactobacillus* Isolates on 3-Hydroxy-3-Methylglutaryl-CoA Reductase and Low-Density Lipoprotein Receptor Genes in Hepatic Cells

**FIGURE 6:**
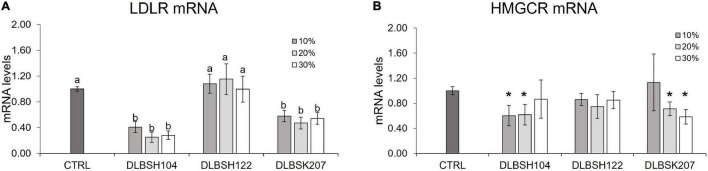
Transcript levels of LDLR **(A)** and HMGCR **(B)** of HepG2 cells treated with CFS of potential *Lactobacillus* strains. HepG2 cells were treated with 10, 20, or 30% CFS of potential *Lactobacillus* strains and cholesterol (70 μg/L) at 37°C for 20 h and then mRNA levels of LDLR and HMGCR was measured by RT-PCR. The result is expressed as mean ± SD of at least three independent experiments. One-way ANOVA or Student’s *t*-test was used and different letters (a, b) or * represents a significant difference with *p*-value < 0.05 compared to the control.

To investigate if Lactobacillus isolates may interfere with the production of HMGCR and LDLR genes, HepG2 cells were given varying doses of probiotics’ CFS and cholesterol for 24 h. Significant downregulation of LDLR and HMGCR was observed when HepG2 cells were treated with CFS from DLBSH104 and DLBSK207 (Figures 6A,B). The addition of 10–20% CFS of DLBSH104 and 20–30% CFS of DLBSK207 exhibited a remarkable reduction (*p* < 0.05) of HMGCR expression, although higher addition of CFS did not show a further reduction of HMGCR levels. In contrast, the addition of 10–30% CFS of DLBSH122 was not able to inhibit the expression of LDLR mRNA (Figure 6A) as well as HMGCR mRNA levels (Figure 6B).

## Discussion

All 11 *Lactobacillus* isolates used in this study demonstrated their ability to remove cholesterol from the media. Among the isolates, DLBSK207, DLBSH104, and DLBSH122 clearly showed a higher ability to remove cholesterol. Some previous studies also observed the elimination of cholesterol from the media by many *Lactobacillus* and *Bifidobacteria* (Miremadi et al., 2014; Choi and Chang, 2015; Ding et al., 2020). Moreover, the ability to remove cholesterol was reported to be dependent on bacterial strain and cell wall composition (Miremadi et al., 2014; Wang et al., 2021). The lower ability to remove cholesterol from media may also be related to a particular bacterial cell composition that prevents cholesterol from attaching (Miremadi et al., 2014). In particular, peptidoglycan isolated from 2 isolates that showed tolerance to bile and had high sodium taurocholate deconjugating abilities—*L. gasseri* SBT0270 and *L. gasseri* SBT0274—was reported to have cholesterol-binding activity. However, the specific composition of peptidoglycan that could bind cholesterol from those isolates is still unknown. Additionally, the different ability to reduce cholesterol in the growth media of 9 strains of *L. helveticus* was revealed by Wang et al. (2021). In that study, the presence of ox bile in media increased the cholesterol removal capability of most strains. Several studies also suggested that the conversion of cholesterol to less soluble metabolites such as 5β-coprostanol and coprostanone leads to a reduction of intestinal cholesterol absorption and an increased elimination in feces (Reis et al., 2017; Bhat and Bajaj, 2019).

This study observed a slight growth reduction of all *Lactobacillus* isolates in the presence of bile acid (ox gall). This condition was also reported in other potential cholesterol-lowering probiotics such as *L. plantarum* EM, *L. sakei* DC1, and *L. acidophilus* ATCC 43121 (Choi and Chang, 2015). One possible explanation of growth reduction in the presence of ox gall is the production of a highly toxic compound called hydrogen sulfide due to taurine metabolism. Taurine itself is produced during deconjugation of taurocholic acid, a bile acid present in ox gall (Bhat and Bajaj, 2019).

BSH activity is another essential factor in choosing an isolate with cholesterol-lowering properties (Tsai et al., 2014). Besides, BSH activity also contributes to the ability of bacteria to survive and colonize in the lower small intestine. From 11 *Lactobacillus* isolates used in this study, two isolates showed strong BSH activity (DLBSH122 and DLBSH131), three isolates showed medium BSH activity (DLBSH113, DLBSH235, and DLBSK207), 1 showed weak BSH activity (DLBSH104), and 5 isolates showed no BSH activity (DLBSL101, DLBSL102, DLBSL103, DLBSA201, and DLBSA202). In general, high BSH activity is related to high bile salt deconjugation abilities (Huang et al., 2019). The deconjugation of bile salt into cholic acid and amino acids such as glycine and taurine leads to the distraction of cholesterol micelle formation, thus reducing lipid absorption in the intestinal tract (Bhat and Bajaj, 2019). In addition, deconjugation of bile salt suppressed the transcription of the 7-α-hydroxylase enzyme responsible for catalyzing bile acid synthesis from cholesterol (Bhat and Bajaj, 2019).

In this current study, we demonstrated that the ability of distinct Lactobacillus isolates to grow in media supplemented with cholesterol and bile salt was not always positively related to BSH activity. Isolates with high BSH activity, such as DLBSH131, showed a high log reduction of *Lactobacillus* growth, while low BSH activity isolates such as DLBSH104 were proved to be more resistant. In agreement with our finding, Moser and Savage (2001) previously reported that BSH activity was unrelated to probiotics’ bile salts resistance ability (Moser and Savage, 2001). Moreover, the protective effect of several ATP-dependent pumps that pump out bile salt in *lactobacilli* is also responsible for improving bile salt cell resistance (Dec et al., 2017; Horackova et al., 2020). Genetic resistance acquired adaptation to surrounding conditions, alteration of the cell wall, and difference in transcription and translation gene related to bile salt resistance are also considered factors that contribute to the growth ability of bacterial cells (Sumeri et al., 2010; Horackova et al., 2020).

*L. helveticus* DLBSH104, *L. plantarum* DLBSH122, and *L. plantarum* DLBSK207 were selected for further experiments based on their ability to remove cholesterol, BSH activity, and ability to grow in the presence of bile salts and cholesterol. Then, we examined their ability to remove cholesterol in a non-growing state (resting or heat-killed) to confirm that their cell surface can bind cholesterol. The result revealed that the growing state of prospective isolates could significantly remove cholesterol from the media although resting and heat-killed cells were still able to remove cholesterol to a lesser degree. Furthermore, the ability of resting cells DLBSH122 and DLBSK207 to reduce cholesterol was significantly higher than dead cells, while the ability of resting and heat-killed DLBSH104 to remove cholesterol was not differ. This finding suggests that cholesterol may be assimilated and incorporated into DLBSH122 and DLBSK207 cell membrane during the metabolic process. Likewise, *Lactobacillus* species including *L*. *acidophillus* ASCC 1520 (Miremadi et al., 2014), *L. rhamnosus* ASCC 1520 (Miremadi et al., 2014), and *L*. *plantarum* EM (Choi and Chang, 2015) have also been found to remove cholesterol in the non-growing state, implying that these strains might be employed as a cholesterol-lowering agent. We postulate that cellular metabolic activities may contribute to our isolates’ ability to remove cholesterol since the cholesterol-lowering ability of those isolates in the growing state was significantly higher than in the non-growing state. Therefore, DLBSH104, DLBSH122, and DLBSK207 in a growing condition are preferable to acquire the most optimal anti-hypercholesterolemic effect.

SEM observation was used to visualize the changes of isolates’ cell surface when they were incubated with cholesterol and bile salt. We found that DLBSH104 is able to bind cholesterol on their cell surface both in a growing and in a non-growing state, but the cell shape between the control and treated group is relatively similar (Figure 3). It is believed that the ability of cholesterol to bind on bacterial cells is through physical events, and only certain amino acids that arrange cell wall peptidoglycan have better capability to bind cholesterol (Kimoto-Nira et al., 2007). Usman and Hosono had examined the variation of cholesterol-lowering ability from 28 *Lactobacillus gasseri* strains, and peptidoglycan isolated from 2 superior strains, SBT0270 and SBT0274, was discovered to be able to bind cholesterol in the media (Hosono, 1999; Pato and Hosono, 1999). Nevertheless, lower cholesterol binding capacity of isolated peptidoglycan than peptidoglycan bound to intact cells was noticed in both strains (Hosono, 1999). It was hypothesized that lower cholesterol binding capacity in isolated peptidoglycan is related to the loss of surface polysaccharides during isolation with TCA (Hosono, 1999). Unfortunately, no study has been done to find out the specific peptidoglycan composition or structure that is responsible for cholesterol adhesion. A study by Li and Papadopoulos (1998) revealed a specific amino acid motif that provides a cholesterol binding site called CRAC (Cholesterol Recognition Amino Acid Consensus) with the following sequence: leucine/valine-(X_1–5_)-tyrosine-(X_1–5_)-arginine/lysine, where X is any amino acid (Li and Papadopoulos, 1998). Direct interaction between arginine and tyrosine with cholesterol was suggested since recombinant *E. coli* that contained CRAC sequence but carried a mutant of tyrosine or arginine did not show any ability to accumulate cholesterol (Li and Papadopoulos, 1998). Moreover, a newer study also suggests the importance of tyrosine to form a H-bond with the OH group of cholesterol (Epand et al., 2010). Another cholesterol binding domain called CARC [inverted CRAC, arginine/lysine-(X_1–5_)-tyrosine/phenylalanine-(X_1–5_)-leucine/valine] was also found more recently (Baier et al., 2011). Interestingly, an absolute requirement of tyrosine can be replaced by phenylalanine that interacts with cholesterol through the stacking arrangement between the aromatic ring of the amino acid with one of the sterane rings of cholesterol (Fantini and Barrantes, 2013).

In contrast with DLBSH104, a slight change of cell shape was observed when DLBSH122 and DLBSK207 were incubated with cholesterol. The apparent cell shape changes in both of our probiotic isolates are probably related to the ability of certain isolates to assimilate and incorporate cholesterol into their cell membrane. In another study, cholesterol was discovered to alter the cell membrane of probiotic isolates through fatty acid composition change (Miremadi et al., 2014). Moreover, the hardening of the *L. acidophillus* ATCC43121 cell membrane due to the assimilation of cholesterol into the cellular membrane improved the membrane cell resistance to lysis by sonication (Noh et al., 1997). Since cholesterol in the media may be incorporated into the cell membrane, the availability of readily absorbed cholesterol in the intestine may be decreased.

We also investigated whether our potential isolates could affect the cholesterol homeostasis regulation in Caco-2 and HepG2 cells. DLBSH104, DLBSH122, and DLBSK207 did not show any toxicity toward Caco-2 cell viability as observed by MTT assay. Therefore, we examined the mRNA expression of cholesterol homeostasis-related genes. In this study, all isolates had successfully suppressed the expression of NPC1L1, although a significant reduction was observed only when the DLBSH104 cell was added. Reduction of NPC1L1 by the addition of our potential isolates is believed to reduce the uptake of cholesterol in the enterocyte of intestinal cells. The reduction of NPC1L1 may be related to the downregulation of SREBP-2 and upregulation of PPARα (Le and Yang, 2019) partly through the activation of the LXR-mediated signaling pathway (Huang and Zheng, 2010). However, our isolates did not prove to suppress the expression of genes related to cholesterol efflux (ABCG5/8) and cholesterol synthesis (HMGCR) in Caco-2 cells.

Hepatic expression of LDL receptor (LDLR) is essential for blood cholesterol regulation. The effect of probiotics on LDLR gene expression was reported to be varied among strains (Kim et al., 2014; Heo et al., 2018; Palaniyandi et al., 2020). *L. fermentum* MJM60397 could upregulate the LDLR levels in the liver of the high-cholesterol diet mice group (Palaniyandi et al., 2020), while *L. plantarum* LRCC5273 treatment did not alter LDLR level in the liver of C57BL/6 mice fed a high-cholesterol diet (Heo et al., 2018). In this study, we demonstrated that CFS from DLBSH104 and DLBSK207 were able to reduce the mRNA expression of LDLR significantly. Hence, our finding is in accordance with Kim et al. (2014) that demonstrated the improvement of lipid metabolism dysregulation in rats fed a high-fat diet supplemented with fermented soymilk *via Lactobacillus plantarum* KCTC10782BP through the significant suppression of LDLR. Additionally, cell-free supernatant of *L. acidophilus* ATCC 43121, *B. Flexus* MCC 2458, *B. Flexus* MCC 2427, and *B. Flexus* MCC 2514 were previously reported to reduce cholesterol levels in the media (Kim et al., 2008; Shobharani and Halami, 2016). Both studies also showed that CFS of all isolates were stable under a variety of heat also in acidic and alkaline conditions (Kim et al., 2008; Shobharani and Halami, 2016). We hypothesized that the downregulation of LDLR expression levels in our study, as well as in others, might be related to the lower availability of cholesterol in the media because of the ability of certain substances in CFS such as peptides to bind with cholesterol. Another possible explanation is the conversion of cholesterol by CFS to other form(s) that are unable to bind LDL receptors. The active component in the CFS of DLBSH104 and DLBSK207 is yet to be investigated.

Besides LDLR mRNA, CFS of both DLBSH104 and DLBSK207 also demonstrated their ability to reduce mRNA levels of HMGCR (an important enzyme that regulates cholesterol synthesis) in HepG2 cells. We proposed that CFS containing short-chain fatty acids of DLBSH104 and DLBSK207 could be responsible for the downregulation of HMGCR level since Bhat and Bajaj suggest that SCFAs produced by LAB probiotics hold an important role in cholesterol metabolism in a host cell (Bhat and Bajaj, 2020). Specifically, propionate was reported to inhibit cholesterol and lipid biosynthesis (Kumar et al., 2012; Tsai et al., 2014) and butyric acid is a powerful inhibitor of HMGCR (Bhat and Bajaj, 2020). A previous study also showed that the suppression of HMGCR at the transcriptional level of HepG2 cells treated with *L. acidophilus* TCCC 11036 could be linked to either NFkB expression reduction or inhibition of NFkB to attach to the promoter region of HMGCR mRNA since a NFkB crucial binding site was discovered on the promoter region of HMGCR (−265 bp) and it has been demonstrated to play an essential role in HMGCR transcription activation (Chen et al., 2016). Moreover, supplementation of soymilk fermented with *Lactobacillus plantarum* KCTC10782BP in high-fat diet rat could significantly suppress the expression of HMGCR through the reduction of SREBP-2, a key regulator protein of cholesterol in cell (Kim et al., 2014). The ability of cell-free supernatant to repress LDLR and HMGCR expression levels suggests that besides direct contact of *Lactobacillus* isolates with cholesterol, metabolites secreted by DLBSH104 and DLBSK207 are sufficient to bring anti-hyperlipidemia effects in host cell.

## Conclusion

In conclusion, our finding identified that *L. helveticus* DLBSH104 and *L. plantarum* DLBSK207 are two outstanding isolates with the best cholesterol-lowering properties among 11 other isolates. Both isolates demonstrated their high capability to remove cholesterol from media, high BSH activity, and resistance in the presence of bile salts and cholesterol in their growth media. The cholesterol-lowering effect in both isolates is attributed to the metabolic process by live cells. Additionally, the presence of *L. helveticus* DLBSH104 and *L. plantarum* DLBSK207 can also be beneficial to host cells by reducing the cholesterol uptake through the downregulation of NPC1L1 in intestinal cells and suppression of genes that are responsible for cholesterol uptake (LDLR) and cholesterol synthesis (HMGCR) in hepatic cells. These 2 isolates might be intended as *Lactobacillus* with anti-hypercholesterolemic effects, while more *in vivo* and human studies are needed.

## Data Availability Statement

The original contributions presented in the study are included in the article/supplementary material, further inquiries can be directed to the corresponding author/s.

## Author Contributions

MK designed the study, performed the experiments, and analyzed the data. AH contributed to the study design and data analysis. RT contributed to the original idea, project supervision, and data analysis. All authors contributed to the manuscript drafting and editing and approved the final version of this manuscript.

## Conflict of Interest

All authors were employed by Dexa Medica. The authors declare that this study received funding from Dexa Medica, but the funder had no role in study design, data collection, analysis, and interpretation.

## Publisher’s Note

All claims expressed in this article are solely those of the authors and do not necessarily represent those of their affiliated organizations, or those of the publisher, the editors and the reviewers. Any product that may be evaluated in this article, or claim that may be made by its manufacturer, is not guaranteed or endorsed by the publisher.

## Abbreviations

BSH: bile salt hydrolase
CFS: cell-free supernatant
CVD: cardiovascular disease
MEM: minimum essential medium
FBS: fetal bovine serum
MRS Broth: de man rogosa and sharpe broth
SEM: scanning electron microscope
PBS: phosphate buffer saline
CFU: colony-forming unit
MTT: 3-(4,5-dimethylthiazol-2-yl)-2,5-diphenyl-2H-tetrazolium bromide
TCA: trichloroacetic acid
CRAC: Cholesterol Recognition/interaction Amino acid Consensus
LDLR: low-density lipoprotein receptor
HMGCR: 3-hydroxy-3-methylglutaryl-CoA reductase
NPC1L1: Niemann-pick C1-like 1
ABCG5: ATP Binding Cassette Subfamily G Member 5
ABCG8: ATP Binding Cassette Subfamily G Member 8
SREBP-2: Sterol regulatory element-binding protein 2
ANOVA: analysis of variance.

## References

[B1] Artham S. M. Lavie C. J. Milani R. V. Ventura H. O. (2008). The obesity paradox: impact of obesity on the prevalence and prognosis of cardiovascular diseases. *Postgrad. Med.* 120 34–41. 18654066 10.3810/pgm.2008.07.1788

[B2] Baier C. J. Fantini J. Barrantes F. J. (2011). Disclosure of cholesterol recognition motifs in transmembrane domains of the human nicotinic acetylcholine receptor. *Sci. Rep.* 1:69. 10.1038/srep00069 22355588 PMC3216556

[B3] Bellosta S. Paoletti R. Corsini A. (2004). Safety of statins: focus on clinical pharmacokinetics and drug interactions. *Circulation* 109(23 Suppl. 1), III50–III57. 10.1161/01.CIR.0000131519.15067.1f 15198967

[B4] Berlian G. Tandrasasmita O. M. Suciptan D. A. S. Tjandrawinata R. R. (2018). Forhidrol, a bioactive fraction of Phaleria macrocarpa(Scheff.) Boerl.,increases reverse cholesterol transport pathway by down-regulation ofcholesteryl ester transfer protein activity. *J. Biol. Res.* 91:6863.

[B5] Bhat B. Bajaj B. K. (2019). Hypocholesterolemic potential of probiotics: concept and mechanistic insight. *J. Exp. Biol.* 57:13.

[B6] Bhat B. Bajaj B. K. (2020). Multifarious cholesterol lowering potential of lactic acid bacteria equipped with desired probiotic functional attributes. *3 Biotech* 10:200. 10.1007/s13205-020-02183-8 32309109 PMC7150668

[B7] Campana R. van Hemert S. Baffone W. (2017). Strain-specific probiotic properties of lactic acid bacteria and their interference with human intestinal pathogens invasion. *Gut. Pathog.* 9:12. 10.1186/s13099-017-0162-4 28286570 PMC5338089

[B8] Chen K. Li S. Chen F. Li J. Luo X. (2016). Regulation of the Lactobacillus Strains on HMGCoA Reductase Gene Transcription in Human HepG2 Cells *via* Nuclear Factor-kappaB. *J. Microbiol. Biotechnol.* 26 402–407. 10.4014/jmb.1507.07086 26528536

[B9] Choi E. A. Chang H. C. (2015). Cholesterol-lowering effects of a putative probiotic strain Lactobacillus plantarum EM isolated from kimchi. *LWT - Food Sci. Technol.* 62 210–217.

[B10] de Melo Pereira G. V. de Oliveira Coelho B. Magalhaes Junior A. I. Thomaz-Soccol V. Soccol C. R. (2018). How to select a probiotic? A review and update of methods and criteria. *Biotechnol. Adv.* 36 2060–2076. 10.1016/j.biotechadv.2018.09.003 30266342

[B11] Dec M. Urban-Chmiel R. Stepien-Pysniak D. Wernicki A. (2017). Assessment of antibiotic susceptibility in Lactobacillus isolates from chickens. *Gut. Pathog.* 9:54. 10.1186/s13099-017-0203-z 28932278 PMC5605976

[B12] Ding Z. Hani A. Li W. Gao L. Ke W. Guo X. (2020). Influence of a cholesterol-lowering strain Lactobacillus plantarum LP3 isolated from traditional fermented yak milk on gut bacterial microbiota and metabolome of rats fed with a high-fat diet. *Food Funct.* 11 8342–8353. 10.1039/d0fo01939a 32930686

[B13] Epand R. M. Thomas A. Brasseur R. Epand R. F. (2010). Cholesterol Interaction with Proteins That Partition into Membrane Domains: an Overview. *Subcell Biochem.* 51 253–278. 20213547 10.1007/978-90-481-8622-8_9

[B14] Fantini J. Barrantes F. J. (2013). How cholesterol interacts with membrane proteins: an exploration of cholesterol-binding sites including CRAC, CARC, and tilted domains. *Front. Physiol.* 4:31. 10.3389/fphys.2013.00031 23450735 PMC3584320

[B15] Ferri N. Corsini A. (2020). Clinical Pharmacology of Statins: an Update. *Curr. Atheroscler. Rep.* 22:26. 10.1007/s11883-020-00844-w 32494971

[B16] Fuentes M. C. Lajo T. Carrión J. M. Cuñé J. (2016). A randomized clinical trial evaluating a proprietary mixture of Lactobacillus plantarum strains for lowering cholesterol. *Mediterr. J. Nutr. Metab.* 9 125–135.

[B17] Heo W. Lee E. S. Cho H. T. Kim J. H. Lee J. H. Yoon S. M. (2018). Lactobacillus plantarum LRCC 5273 isolated from Kimchi ameliorates diet-induced hypercholesterolemia in C57BL/6 mice. *Biosci. Biotechnol. Biochem.* 82 1964–1972. 10.1080/09168451.2018.1497939 30032716

[B18] Horackova S. Vesela K. Klojdova I. Bercikova M. Plockova M. (2020). Bile salt hydrolase activity, growth characteristics and surface properties in Lactobacillus acidophilus. *Euro. Food Res. Technol.* 246 1627–1636.

[B19] Hosono A. (1999). Binding of cholesterol to the cells and peptidoglycan of Lactobacillus gasseri. *Milchwissenschaft* 54 495–498.

[B20] Huang C. H. Ho C. Y. Chen C. T. Hsu H. F. Lin Y. H. (2019). Probiotic BSH Activity and Anti-Obesity Potential of Lactobacillus plantarum Strain TCI378 Isolated from Korean Kimchi. *Prev. Nutr. Food Sci.* 24 434–441. 10.3746/pnf.2019.24.4.434 31915639 PMC6941724

[B21] Huang Y. Zheng Y. (2010). The probiotic Lactobacillus acidophilus reduces cholesterol absorption through the down-regulation of Niemann-Pick C1-like 1 in Caco-2 cells. *Br. J. Nutr.* 103 473–478. 10.1017/S0007114509991991 19814836

[B22] Kartawijaya M. Han H. W. Kim Y. Lee S. M. (2016). Genistein upregulates LDLR levels *via* JNK-mediated activation of SREBP-2. *Food Nutr. Res.* 60:31120. 10.3402/fnr.v60.31120 27211318 PMC4876195

[B23] Khare A. Gaur S. (2020). Cholesterol-Lowering Effects of Lactobacillus Species. *Curr. Microbiol.* 77 638–644. 10.1007/s00284-020-01903-w 32020463

[B24] Khvorova A. (2017). Oligonucleotide Therapeutics - A New Class of Cholesterol-Lowering Drugs. *N. Engl. J. Med.* 376:4–7. 10.1056/NEJMp1614154 28052224

[B25] Kim Y. Whang J. Y. Whang K. Y. Oh S. Kim S. H. (2008). Characterization of the cholesterol-reducing activity in a cell-free supernatant of Lactobacillus acidophilus ATCC 43121. *Biosci. Biotechnol. Biochem.* 72 1483–1490. 10.1271/bbb.70802 18540115

[B26] Kim Y. Yoon S. Lee S. B. Han H. W. Oh H. Lee W. J. (2014). Fermentation of soy milk *via* Lactobacillus plantarum improves dysregulated lipid metabolism in rats on a high cholesterol diet. *PLoS One* 9:e88231. 10.1371/journal.pone.0088231 24520358 PMC3919746

[B27] Kimoto-Nira H. Mizumachi K. Nomura M. Kobayashi M. Fujita T.l.Y. Okamoto T. (2007). Lactococcus sp. as Potential Probiotic Lactic Acid Bacteria. *Jpn. Agric. Res. Q.* 41:181.

[B28] Kopin L. Lowenstein C. J. (2017). Dyslipidemia. *Ann. Intern. Med.* 167 ITC81–ITC96. 29204622 10.7326/AITC201712050

[B29] Kumar M. Nagpal R. Kumar R. Hemalatha R. Verma V. Kumar A. (2012). Cholesterol-Lowering Probiotics as Potential Biotherapeutics for Metabolic Diseases. *Exp. Diabet. Res.* 2012:902917. 10.1155/2012/902917 22611376 PMC3352670

[B30] Le B. Yang S. H. (2019). Effect of potential probiotic Leuconostoc mesenteroides FB111 in prevention of cholesterol absorption by modulating NPC1L1/PPARalpha/SREBP-2 pathways in epithelial Caco-2 cells. *Int. Microbiol.* 22 279–287. 10.1007/s10123-018-00047-z 30810991

[B31] Li H. Papadopoulos V. (1998). Peripheral-type benzodiazepine receptor function in cholesterol transport. Identification of a putative cholesterol recognition/interaction amino acid sequence and consensus pattern. *Endocrinology* 139 4991–4997. 10.1210/endo.139.12.6390 9832438

[B32] Lye H.-S. Rahmat-Ali G. R. Liong M.-T. (2010). Mechanisms of cholesterol removal by lactobacilli under conditions that mimic the human gastrointestinal tract. *Int. Dairy J.* 20 169–175.

[B33] Mann G. V. (1974). Studies of a surfactant and cholesteremia in the Maasai. *Am. J. Clin. Nutr.* 27 464–469. 10.1093/ajcn/27.5.464 4596028

[B34] McFarland L. V. Evans C. T. Goldstein E. J. C. (2018). Strain-Specificity and Disease-Specificity of Probiotic Efficacy: a Systematic Review and Meta-Analysis. *Front. Med.* 5:124. 10.3389/fmed.2018.00124 29868585 PMC5949321

[B35] Michael D. R. Moss J. W. Calvente D. L. Garaiova I. Plummer S. F. Ramji D. P. (2016). Lactobacillus plantarum CUL66 can impact cholesterol homeostasis in Caco-2 enterocytes. *Benef. Microbes* 7 443–451. 10.3920/BM2015.0146 26839071

[B36] Miremadi F. Ayyash M. Sherkat F. Stojanovska L. (2014). Cholesterol reduction mechanism and fatty acid composition of cellular membranes of probiotic Lactobacilli and Bifidobacteria. *J. Funct. Foods* 9 295–305.

[B37] Moser S. A. Savage D. C. (2001). Bile salt hydrolase activity and resistance to toxicity of conjugated bile salts are unrelated properties in lactobacilli. *Appl. Environ. Microbiol.* 67 3476–3480. 10.1128/AEM.67.8.3476-3480.2001 11472922 PMC93046

[B38] Najib S. Z. Fachri W. Sauriasari R. Elya B. Tjandrawinata R. (2018). Cholesterol-Lowering Effects of Extract from Garcinia daedalanthera in Hyperlipidemic Rats. *Pharmacogn. J.* 10 1125–1128.

[B39] Noh D. O. Kim S. H. Gilliland S. E. (1997). Incorporation of cholesterol into the cellular membrane of Lactobacillus acidophilus ATCC 43121. *J. Dairy Sci.* 80 3107–3113. 10.3168/jds.S0022-0302(97)76281-7 9436091

[B40] Palaniyandi S. A. Damodharan K. Suh J. W. Yang S. H. (2020). Probiotic Characterization of Cholesterol-Lowering Lactobacillus fermentum MJM60397. *Probiotics Antimicrob. Proteins* 12 1161–1172. 10.1007/s12602-019-09585-y 31432401

[B41] Pato U. Hosono A. (1999). Bile tolerance, taurocholate deconjugation, and binding of cholesterol by Lactobacillus gasseri strains. *J. Dairy Sci.* 82 243–248. 10.3168/jds.S0022-0302(99)75229-X 10068945

[B42] Pinzon R. T. Tjandrawinata R. R. Wijaya V. O. Veronica V. (2021). Effect of DLBS1033 on Functional Outcomes for Patients with Acute Ischemic Stroke: a Randomized Controlled Trial. *Stroke Res. Treat.* 2021 5541616. 10.1155/2021/5541616 33927846 PMC8049819

[B43] Reis S. A. Conceição L. L. Rosa D. D. Siqueira N. P. Peluzio M. C. G. (2017). Mechanisms responsible for the hypocholesterolaemic effect of regular consumption of probiotics. *Nutr. Res. Rev.* 30 36–49. 27995830 10.1017/S0954422416000226

[B44] Shobharani P. Halami P. M. (2016). In vitro evaluation of the cholesterol-reducing ability of a potential probiotic Bacillus spp. *Ann. Microbiol.* 66 643–651.

[B45] Sinaga W. S. L. B. Ismaya W. T. Retroningrum D. S. Tjandrawinata R. R. Suhartono M. T. (2020). Peptides Hydrolysate Derived from Collagen of Snakehead Murrel (Channa striata) Skin Demonstrate Anti-cholesterol and Anti-oxidant activities. *Hayati J. Biosci.* 27 136–141.

[B46] Sivamaruthi B. S. Kesika P. Chaiyasut C. (2019). A Mini-Review of Human Studies on Cholesterol-Lowering Properties of Probiotics. *Sci. Pharm.* 87:26.

[B47] Sumeri I. Arike L. Stekolstsikova J. Uusna R. Adamberg S. Adamberg K. (2010). Effect of stress pretreatment on survival of probiotic bacteria in gastrointestinal tract simulator. *Appl. Microbiol. Biotechnol.* 86 1925–1931. 10.1007/s00253-009-2429-2 20107984

[B48] Tandrasasmita O. M. Berlian G. Tjandrawinata R. R. (2021). Molecular mechanism of DLBS3733, a bioactive fraction of Lagerstroemia speciosa (L.) Pers., on ameliorating hepatic lipid accumulation in HepG2 cells. *Biomed. Pharmacother.* 141:111937. 10.1016/j.biopha.2021.111937 34328120

[B49] Tsai C. C. Lin P. P. Hsieh Y. M. Zhang Z. Y. Wu H. C. Huang C. C. (2014). Cholesterol-lowering potentials of lactic acid bacteria based on bile-salt hydrolase activity and effect of potent strains on cholesterol metabolism in vitro and in vivo. *Sci. WorldJ.* 2014:690752. 10.1155/2014/690752 25538960 PMC4235975

[B50] Vaughan M. Murphy M. Brendan M Buckley D. (1996). Statins do more than just lower cholesterol. *The Lancet* 348 1079–1082. 10.1016/S0140-6736(96)05190-2 8874463

[B51] Wang G. Chen X. Wang L. Zhao L. Xia Y. Ai L. (2021). Diverse conditions contribute to the cholesterol-lowering ability of different Lactobacillus plantarum strains. *Food Funct.* 12 1079–1086. 10.1039/d0fo02073g 33367350

[B52] Wang L. Guo M. J. Gao Q. Yang J. F. Yang L. Pang X. L. (2018). The effects of probiotics on total cholesterol: a meta-analysis of randomized controlled trials. *Medicine* 97:e9679. 10.1097/MD.0000000000009679 29384846 PMC5805418

[B53] Yang F. Chen G. Ma M. Qiu N. Zhu L. Li J. (2018). Fatty acids modulate the expression levels of key proteins for cholesterol absorption in Caco-2 monolayer. *Lipids Health Dis.* 17:32. 10.1186/s12944-018-0675-y 29463265 PMC5819267

